# Impact of different front-of-package labeling models on food choices among residents in Shanghai, China: a randomized controlled trial

**DOI:** 10.3389/fnut.2025.1594807

**Published:** 2025-07-04

**Authors:** Zhuo Sun, Xiao Chen, Jiangyue Yu, Wei Lu, Zehuan Shi, Kunyi Huang, Liping Shen, Wenqing Ma, Shupeng Mai, Qi Song, Zhengyuan Wang, Jiajie Zang

**Affiliations:** ^1^Department of Nutrition and Health, Division of Health Risk Factors Monitoring and Control, Shanghai Municipal Center for Disease Control and Prevention, Shanghai, China; ^2^Shanghai Putuo District Center for Disease Control and Prevention, Shanghai, China; ^3^School of Public Health, Fudan University, Shanghai, China; ^4^School of Public Health, Shanghai University of Traditional Chinese Medicine, Shanghai, China

**Keywords:** front-of-package labeling, prepackaged foods, NC, randomized controlled trial, China

## Abstract

**Background:**

Increased convenience food consumption poses considerable public health concerns because of its high oil, salt, and sugar content. The World Health Organization endorses front-of-pack labeling (FOPL) as a strategy to enhance consumer choice toward healthier options. Despite their global adoption, the efficacy of various FOPL systems in China’s unique market remains unexplored. Here, we evaluated the impact of different FOPL models on the food purchasing behaviors of Shanghai residents.

**Methods:**

This randomized controlled trial included 7,346 respondents, who were randomly assigned to one of four FOPL groups: Nutri-Choice (NC), Nutrition Information Panel (NIP), Comprehensive Nutri-Choice (CNC), and Warning Label (WL). An online questionnaire was used to collect the respondents’ demographic profiles. The sensory perceptions, nutritional ranking ability, and intention to purchase healthy foods of the respondents were evaluated using a simulated shopping experiment.

**Results:**

In total, 88.03% of the respondents supported FOPL use in China. Respondents completed the NC and WL questionnaires more quickly than the CNC or NIP questionnaire, with the NIP questionnaire taking the longest to complete (*Z* = 24.209, df = 3, *p* < 0.001). Sensory perception total approval rates were significantly higher for NC (57.01%), CNC (58.04%), and WL (57.77%) than for NIP (43.33%; *χ^2^* = 112.958, df = 3, *p* < 0.001). Similarly, the total accuracy rate in the nutritional ranking was significantly higher for NC (69.94%) and CNC (72.45%) than for WL (55.44%) and NIP (32.99%; *χ^2^* = 737.823, df = 3, *p* < 0.001). The purchasing intentions were the healthiest for CNC (83.10%), followed by NC (80.09%)—with both significantly outperforming NIP (75.11%) and WL (73.42%; *χ^2^* = 63.360, df = 3, *p* < 0.001).

**Conclusion:**

NC and CNC potentially improve consumers’ understanding of packaged food nutritional data, encouraging healthier purchasing decisions. Our results provide a scientific rationale for formulating and implementing an impactful FOPL initiative in China.

## Introduction

In recent years, consumers have been favoring prepackaged foods because of their convenience and quick preparation, resulting in a rapid growth in their consumption (1). From 1999 to 2012, the per capita retail value of prepackaged foods increased in developing countries, such as China, Vietnam, and Thailand (a 192%, 166%, and 65% increase, respectively) (2). Simultaneously, ultraprocessed foods (mostly prepackaged foods) accounted for a large proportion of daily energy intake in developed countries, such as United States and Canada (57.9% and 45.0%, respectively) (3). Although consumption of prepackaged foods remains lower in China than in developed countries, its rising trend is noteworthy. The 2019 data indicated a 7% increase in the caloric share of ultraprocessed food consumption since 2013, demonstrating its persistent upward trajectory in China (3). However, the nutritional quality of these ultraprocessed foods varies widely. Compared with unpackaged foods, prepackaged foods often have higher added-sugar, sodium, and saturated- and trans-fat contents. Moreover, increased consumption of prepackaged foods may lead to diet-related chronic noncommunicable diseases including obesity, 2 diabetes, and cardiovascular disease (4). As prepackaged foods have become a crucial component of individual diets (5), communicating nutritional information effectively to consumers through clear, concise food labeling remains imperative. This can allow consumers to discern the nutritional value of prepackaged foods they purchase quickly.

In China, nearly all prepackaged foods have been required to display a NIP since 2013; it must at least include the energy, protein, fat, carbohydrates and sodium contents in a fixed weight or volume according to *General Principles for Nutritional Labels of Prepackaged Foods* (6). However, these food labels are often positioned on the back or side of food packages, making them prone to being overlooked. Some studies have pointed out that even after noticing these labels, many consumers cannot interpret the information on these labels accurately. Consequently, in practice, these labels have a limited impact on guiding food choices among consumers (7).

To guide consumers in distinguishing the nutritional content of prepackaged foods and facilitate the improvement of unhealthy eating habits efficiently and conveniently, the World Health Organization (WHO) has recommended the adoption of simple, intuitive FOPL (8). As of March 2024, 11 countries had adopted mandatory FOPL, 4 countries implemented both mandatory and voluntary FOPLs and 28 countries implemented voluntary (9). Furthermore, an increasing number of countries are considering it a valuable tool for national public health initiatives. Globally, FOPL types can be primarily divided into two categories: non-interpretative designs, such as Guideline Daily Amount (GDA) and Reference Intakes (RI), and interpretative designs, which include three subtypes—nutrient-specific designs like WL and Multiple Traffic Light (MTL), summary designs like Nutri-Score (NS) and Health Star Rating (HSR), and positive endorsement designs like Pick the Tick) (10). Of these, noninterpretative designs offer detailed nutritional insights but can be challenging to comprehend, thus hindering quick consumer decisions (11, 12). NIP, currently implemented in China, is also a noninterpretative design. WL alerts consumers to foods high in nutrients such as sodium, sugar, and fats, and despite being effective, these labels lack other relevant information (i.e., protein, vitamins, calcium, etc.) (12, 13). On the other hand, MTL may cause confusion related to information overload, due to its bright colors and ratings for each target nutrient (14). Summary designs have a wide range of applications, and their ratings based on overall nutritional quality are easy for consumers to understand and choose from quickly. However, they only provide limited nutritional details; thus, their efficacy in discouraging consumers from buying unhealthy options warrants enhancement (4, 12, 15, 16). Positive endorsement designs can aid consumers to identify healthier options by endorsing products that meet predefined nutritional criteria; however, only a few products meeting healthy criteria are labeled, and the information provided is inadequate for product comparison (5). In general, each FOPL model has pros and cons.

Several teams in China have developed FOPL tailored specifically for the country’s market; however, the most effective FOPL model for guiding consumers during their shopping remains unknown (14, 17, 18). Moreover, the “Healthy China Action Plan (2019–2030)”—a national health intervention strategy issued by the State Council of the People’s Republic of China—explicitly states the need to promote the use of FOPL information on food packages actively (19). Therefore, we selected four types of FOPL by integrating the most popular FOPL internationally and those previously developed by Chinese research teams, and subsequently conducted a population survey in Shanghai in 2024 to comprehend their preferences for various FOPL models. We also assessed the influence of these FOPL models on residents’ shopping in China in terms of four dimensions: attitudes toward FOPL, sensory perception of specific FOPL models, effects of specific FOPL models on nutritional ranking ability, and intention to purchase healthy foods, aimed to identify. Which type of FOPL resonated with consumers in China.

## Methods

### Sample size calculation

We adopted a randomized controlled trial design, using online self-reported questionnaire to collect data. To calculate the sample size for each group, we used the sample size calculation formula for multiple group comparisons of randomized controlled trials (RCTs): n = (Z_*α*/2_ + Z*_β_*)^2^ × ((*p*_max_(1 − *p*_min_) + *p*_min_(1 − *p*_max_))/(p_max_ − p_min_)^2^ × 1/g. In our pilot study, *p*_max_ = 0.82 and *p*_min_ = 0.74. We defined the two-sided significance level α = 0.05, 1 − β = 0.8, Zα/2 = 1.96, Z_β_ = 0.84. Considering a 20% dropout rate, we planned to recruit 133 respondents in each group. Our study had four respondent groups by FOPL models, each divided into two levels by sex and four levels by age; therefore, our study required at least 133 × 4 × 2 × 4 = 4,256 participants.

The study was approved by the Ethics Committee of Shanghai Center for Disease Control and Prevention (KY-2024-43) and was performed in accordance with the Declaration of Helsinki. Before participating in the survey, for adult participants, informed consent was obtained directly from the individuals themselves, for minors, informed consent was provided by their legal guardians.

### Respondent recruitment and grouping

From April to May 2024, 400 community residents were randomly recruited from each of the 16 districts in Shanghai. Respondents should use a smartphone for 3 + years or were proficient in using a smartphone. Exclusion criteria included the presence of cognitive disorders, color blindness and color weakness.

The survey was administered as an online self-reported questionnaire to be filled out after scanning a code, completed, and then submitted. After they scanned the code, respondents were randomly assigned to one of the four FOPL model groups. Block randomization was used to assign respondents to groups, which resulted in eight blocks based on age and sex. After a block reached its capacity, it was excluded from further assignment. Specifically, the sample was stratified by age group and gender, the 18–40, 40–60, and >60-year age groups included 80 respondents each, whereas the <18-year age group included 160 respondents, and equal gender ratio.

**Figure 1:**
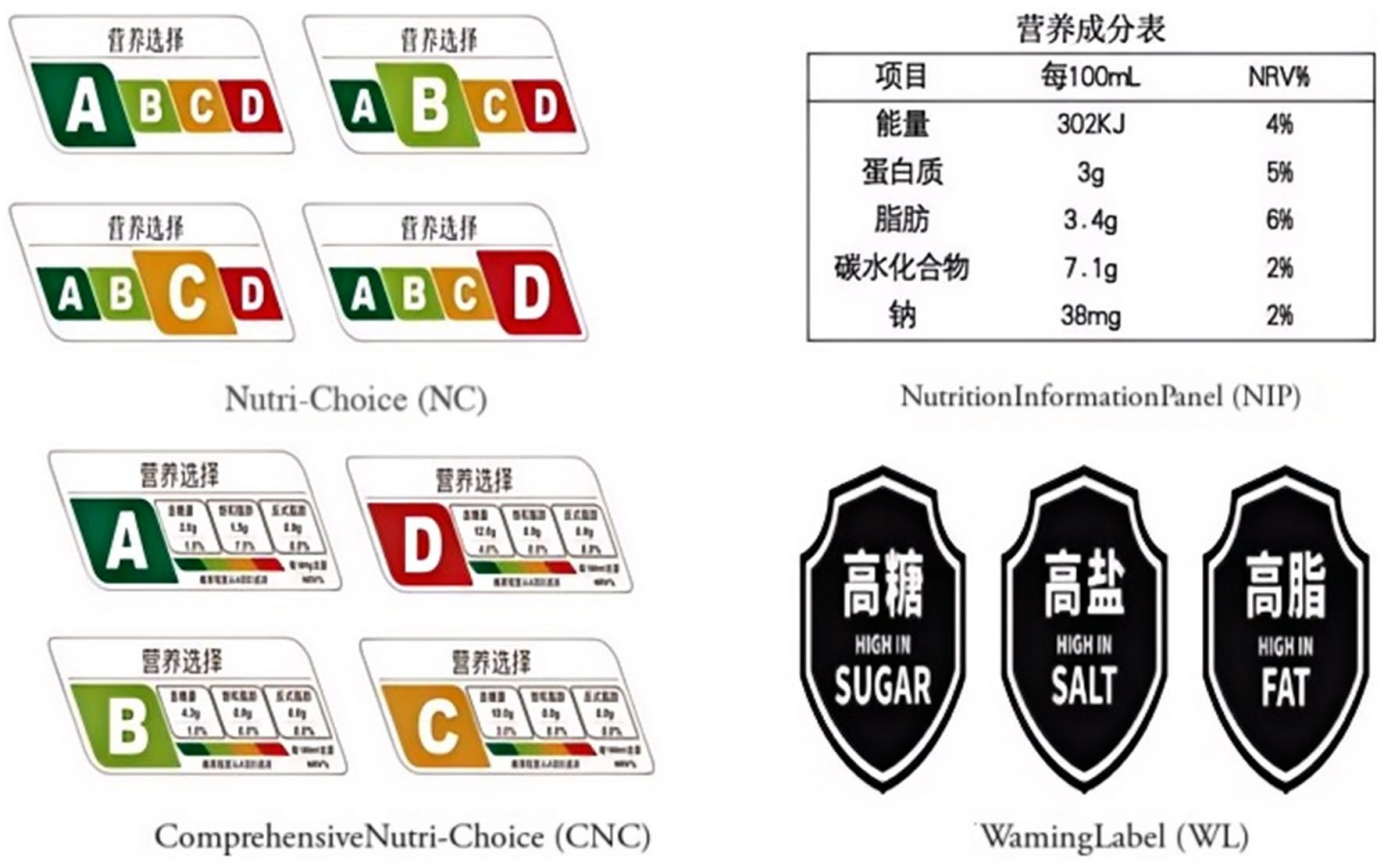
Four models of FOPL.

We set four FOPL model groups: (1) NC. This was China’s first voluntary summary grade-assessment labeling, initiated by a government-led pilot program in Shanghai. NC only enlarges the size of the corresponding ABCD letter grading based only on the comprehensive evaluation results of multiple nutrient contents, including non-milk extrinsic sugars, non-sugar sweeteners, saturated- and trans-fat. The bigger prominent letter represents food grading, and the base color of the letter represents whether they are healthy: green and light green represent health, orange and red are relatively unhealthy and particularly unhealthy. (2) NIP. It is currently used in China as a back-of-pack labeling and provides detailed information regarding energy, protein, fat, carbohydrate, and sodium contents and their percent nutrient reference values. In this study, we placed it prominently on the front of the food package to enhance comparability. (3) CNC. This FOPL had specific nutritional information adapted from NC. It displayed ABCD letter and color grading, along with the comprehensive nutrient contents of sugar, saturated- and trans-fat and their percentages for daily recommended intake. (4) WL. This label, designed by the Chinese Center for Disease Control and Prevention (18), consists of three black icons representing high sugar, salt, and fat contents. In our simulated shopping experiment, products were assigned 0 to 3 icons of WL on the basis of their actual nutritional attributes. Figure 1 illustrates specific FOPL models.

### Questionnaire and data interpretation

In the current study, we developed a questionnaire in accordance with the WHO’s FOPL guiding principles and framework manual (20). Our survey questionnaires covered general demographic details, attitudes toward FOPL policy implementation, sensory perceptions regarding various FOPL models, the impact of specific FOPL models on objective nutritional ranking ability, and purchase intentions across five food categories (i.e., fruit juice, potato chips, soda biscuits, instant noodles, and yoghurt). Although each questionnaire featured a different FOPL model, the question design was consistent among all groups, which included sensory perceptions, the impact on objective nutritional ranking ability, and purchase intentions across five food categories.

Education status was divided into three categories: junior high school or lower, high school (general/vocational/secondary/technical school) or junior college, undergraduate and above. Average monthly earnings’ categories were as follows: Less than 2,600 yuan, 2,600–6,000 yuan, 6,000 yuan and above.

Job title refers to the level of professional technical level, ability, and achievement of professional and technical personnel. In this study, Job title divided into four levels: none, junior, intermediate, and senior.

Body mass index (BMI) is a statistical index using a respondent self-reported weight and height. BMI = weight (in kg)/ height^2^ (in m^2^). These classifications for Body mass index (BMI) are in use by the World Health Organization (WHO): Underweight - BMI under 18.5 kg/m^2^, Normal - BMI greater than or equal to 18.5 to 24.9 kg/m^2^, Overweight–BMI greater than or equal to 25 to 29.9 kg/m^2^, Obesity–BMI greater than or equal to 30 kg/m^2^. For children (6 to 18 years old), the method to derive BMI use WHO references for school-age children and adolescents (21).

#### Sensory perceptions regarding various FOPL models

This section was designed to gauge respondents’ perceptions of their assigned FOPL models, and included eight questions:

1. This FOPL leaves a strong impression on me.
2. I prefer this style of FOPL.
3. I find the information on this FOPL trustworthy.
4. This FOPL makes me consider the conveyed information.
5. This FOPL prompts health-related thoughts in my mind.
6. With this FOPL, I can easily choose food.
7. This FOPL influences my food purchasing decisions.
8. I am likely to discuss this FOPL with others next week.

Each item was scored on a 5-point scale, ranging from 1 (*Strongly disagree*) to 5 (*Strongly agree*). For each item, the responses 4 (*Agree*) or 5 (*Strongly agree*) were considered to indicate approval. Respondents who approved all items were considered to have complete approval of the FOPL’s sensory aspects. The higher the proportion of approvals, the more favorable was the respondents’ views regarding FOPL.

#### Effect of specific FOPL models on nutritional ranking ability and intention to purchase healthy foods

We divided the section of the simulated shopping questionnaire into two sections designed based on WHO research guidance, each evaluating nutritional ranking ability and intention to purchase healthy foods (21). Each section addressed five questions pertaining to commonly consumed and frequently consumed food groups: fruit juice, potato chips, soda biscuits, instant noodles, and yoghurt. In each question, three food items with highly similar packaging, identical pricing, and no brand identification were presented in a random order. The respondents were asked to rank the foods based on their specific FOPL, with at least two grading levels for NC and CNC and at least one warning model for WL. For instance, the respondents were asked, “Please rank the healthiness of three models of yoghurt, from the highest to lowest, according to your own judgment.”

In Section 1, each question was considered correct if the ranking adhered to the FOPL principles (the correct ranking of fruit juice was “321”or “231”; the correct ranking of potato chips was “321”or “231”; the correct ranking of soda biscuits was “321”; the correct ranking of instant noodles was “123”; the correct ranking of yoghurt was “321”). The perfect ranking rate was defined as the number of respondents who correctly ranked at least three questions divided by the total respondent count. In section 2, alignment with the healthiest choice according to the FOPL hierarchy indicated the intention to purchase healthy foods (the healthiest choice of fruit juice was “3” or “2”; the healthiest choice of potato chips was “3” or “2”; the healthiest choice of soda biscuits was “3”; the healthiest choice of instant noodles was “1”; the healthiest choice of yoghurt was “3”). The rate of purchasing healthy foods was defined as the number of respondents who chose the healthiest option on at least three questions divided by the total respondent count.

### Quality control

Each questionnaire included this quality control question: “Please select ‘Neither agree nor disagree’ for this question.” The options for this question were “A. Strongly disagree,” “B. Disagree,” “C. Neither agree nor disagree,” “D. Agree,” and “E. Strongly agree.” Respondents who selected option C were considered to have met the qualification criteria, whereas questionnaires in which the respondents selected any option other than C were considered invalid.

### Statistical analysis

Data analysis was conducted using SPSS (version 25). Due to the nonnormal distribution of the labeling scores, we used median (*M*) values and their quartiles (P25, P75) for descriptive purposes. The Kruskal–Wallis rank sum test was used for multiple group comparisons, whereas the chi-square test was used for rate comparisons. The significance level was set at *α* = 0.05. It is unclear whether statistical adjustments (e.g., Bonferroni or other corrections) were applied for the multiple pairwise comparisons across food categories and demographic subgroups.

## Results

### General characteristics of survey respondents

In total, 7,346 valid questionnaires were collected, 7,303 of which were included in the study. Of all respondents, 48.39% were male and 51.61% were female. The average respondent age was 35.42 ± 20.31 years. The snacking preference proportions slightly differ among the four groups. In pairwise comparisons, the NC and WL groups completed the questionnaires faster than the CNC and NIP groups (*Z* = 24.209, df = 3, *p* < 0.001); the NIP group required the longest time to finish (Table 1). Other variables did not differ significantly between groups.

**Table 1:** Basic characteristics of survey respondents (%).

**Characteristic**	Label 1: NC (*n* = 1,833)	Label 2: NIP (*n* = 1,828)	Label 3: CNC (*n* = 1,840)	Label 4: WL (*n* = 1,802)	*p* (chi-square test)
Sex					0.354
Male	46.92	48.69	47.83	49.78	
Female	53.08	51.31	52.17	50.22	
Age (years)					0.818
[6,18)	37.64	36.32	35.49	36.40	
[18, 40)	23.40	23.25	23.37	24.36	
[40,60)	21.33	21.06	22.12	20.20	
≥60	17.62	19.37	19.02	19.03	
Education					0.092
Junior high school or lower	32.02	34.08	35.98	35.74	
High school (general/vocational/secondary/technical school) or junior college	39.44	37.75	35.71	35.35	
Undergraduate and above	39.44	37.75	35.71	35.35	
Average monthly earnings after taxes* (CNY¥)					0.701
Less than 2,600	9.81	9.70	10.98	9.38	
2,600–6,000	40.05	41.52	42.18	42.29	
6,000 and above	50.14	48.78	46.85	48.33	
Occupation					0.803
Intellectual	16.97	18.11	17.66	17.92	
Manual labor	43.64	44.47	45.22	43.62	
Student	39.39	37.42	37.12	38.46	
Job title					0.683
None	68.95	66.35	68.02	66.64	
Junior	13.05	14.25	14.61	14.70	
Intermediate	15.93	17.31	15.21	15.69	
Senior	2.07	2.10	2.16	2.98	
Engaged in nutrition-, food-, or medicine-related industries					0.735
Yes	9.55	10.39	10.27	9.54	
No	90.45	89.61	89.73	90.46	
Body mass index					0.187
Underweight	4.69	4.21	4.46	4.05	
Normal	66.18	64.06	64.57	68.53	
Overweight	23.73	25.66	25.05	21.59	
Obesity	5.40	6.07	5.92	5.83	
At least one serious disease (e.g., diabetes, anaemia, thyroid disorder, and heart disease)					0.690
Yes	3.87	4.05	4.62	4.05	
No	96.13	95.95	95.38	95.95	
Weight loss/shaping					0.808
Yes	10.64	10.18	9.62	10.43	
No	89.36	89.82	90.38	89.57	
Snacking preference					0.039
Like	37.70	37.91	37.39	35.85	
Neither like nor dislike	43.48	41.03	43.48	41.23	
Dislike	18.82	21.06	19.13	22.92	
Snacking frequency					0.065
Daily	10.04	11.16	11.30	10.21	
4–6 times/week	26.57	24.34	23.37	24.36	
1–3 times/week	45.17	43.71	46.25	43.45	
Less than 1 time/week	18.22	20.79	19.08	21.98	
Questionnaire completion time (median and IQR)	217.00(143.00, 339.00)^a^	238.00(158.00, 387.00)^b^	231.00(154.00, 368.75)^c^	208.00(138.00, 336.00)^a^	<0.001

*These data do not include students; the remaining total number is 4521. ^a, b, c, d^The same letter appearing for two groups denotes the absence of significant between-group differences, whereas different letters for different groups denote the presence of significant between-group differences (*p* < 0.05).

### Factors influencing respondents’ attitudes toward FOPL policy

In total, 88.03% of the respondents supported the introduction of the FOPL policy in China, whereas only 0.93% were against it (Supplementary Table S1). Different subgroups of sex, education, income, class of position, snacking frequency, and living with children demonstrated a mild change in the support rate, of which the lowest was 84.74% in respondents snacking most frequently.

### Respondents’ sensory perceptions of specific FOPL models

Sensory perceptions regarding specific FOPL models were evaluated in terms of eight dimensions to quantify the overall acceptance and impact of each FOPL model. The total approval rates for NC (57.01%), CNC (58.04%), and WL (57.77%) were significantly elevated compared with those showing NIP on the front-of-pack (43.33%; *χ^2^* = 112.958, df = 3, *p* < 0.001; Table 2). In the stratified analyses, most factors, except underweight, senior job title, or presence of serious disease, were significant (all *p* < 0.05; Supplementary Table S2).

**Table 2:** Respondent FOPL approval rates in terms of sensory perceptions in different FOPL model groups (%).

Category	NC (*n* = 1,833)	NIP (*n* = 1,828)	CNC (*n* = 1,840)	WL (*n* = 1,802)	*p*
Total	57.01^a^	43.33^b^	58.04^a^	57.77^a^	<0.001
Impression	79.92^a^	63.73^b^	81.36^a^	83.90^c^	<0.001
Likability	76.65^a^	61.87^b^	79.18^a^	79.08^a^	<0.001
Reliability of information	75.01^a^	69.64^b^	77.45^a,c^	77.98^c^	<0.001
Thought provocation	74.14^a^	67.40^b^	76.63^a,c^	78.86^c^	<0.001
Health association	73.92^a^	65.65^b^	75.65^a^	76.08^a^	<0.001
Ease of selection	75.94^a^	62.20^b^	77.55^a^	78.19^a^	<0.001
Influence on purchase	75.40^a^	64.11^b^	76.03^a^	78.80^c^	<0.001
Ability to stimulate discussion	70.70^a,b^	59.14^c^	69.84^b^	72.92^a^	<0.001

^a, b, c, d^The same letter appearing for two groups denotes the absence of significant between-group differences, whereas different letters for different groups denote the presence of significant between-group differences (*p* < 0.05).

**Figure 2:**
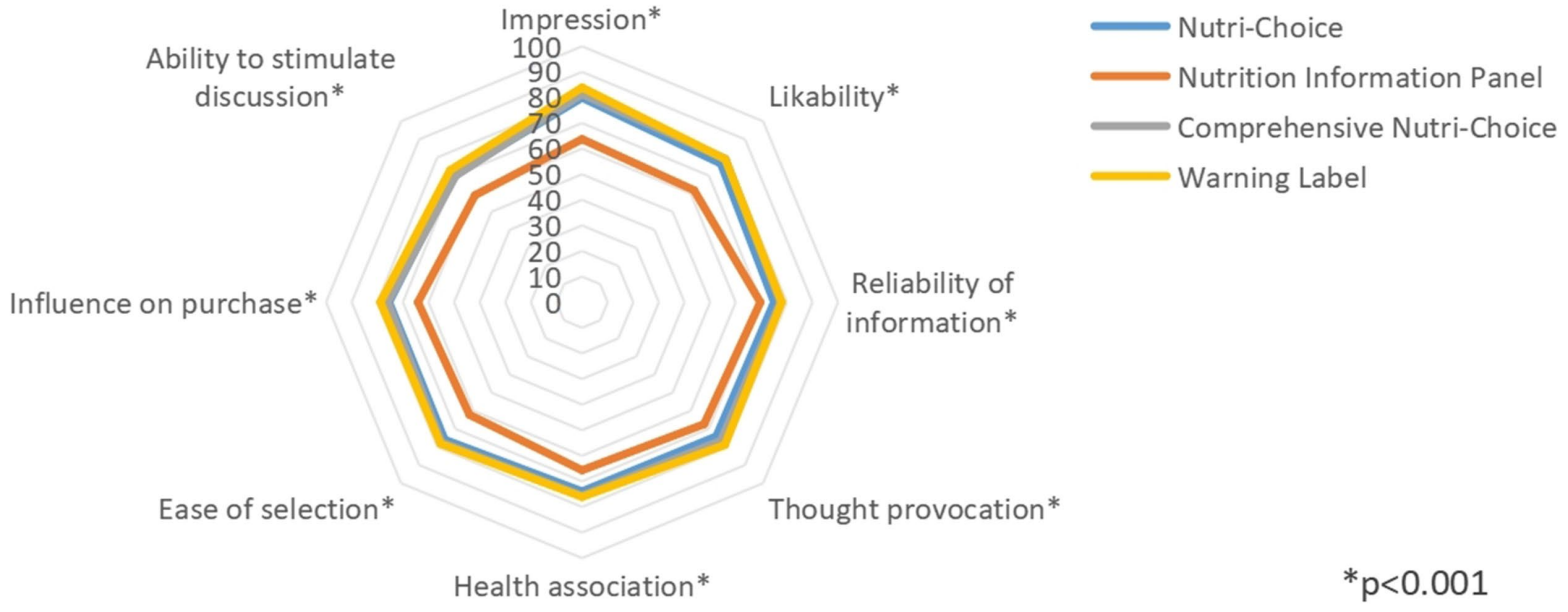
Respondent FOPL approval rates in terms of sensory perceptions in different FOPL model groups.

Assessment across eight dimensions all revealed significant differences (Figure 2 and Table 2). Regarding the two dimensions of impression and impact on purchasing decisions, the pairwise comparison analysis revealed a greater preference for WL over NC and CNC. Moreover, both NC and CNC received significantly more positive feedback than NIP. Regarding preference, health associations, and food purchasing convenience, respondents consistently favored NC, CNC, or WL over NIP. In the assessment of reliability and thought provocation, WL slightly outperformed NC, whereas NIP demonstrated the lowest approval rate. Regarding the ability to stimulate discussion, differences between WL and NC were nonsignificant, but both significantly outperformed CNC and NIP, indicating that the respondents considered graphic nutrition labels novel.

### Effects of different FOPL models on food nutrition ranking

We assessed the effects of specific FOPL models on guiding respondents to rank the healthiness of various foods accurately. As presented in Table 3, the correct ranking rates in the NC (69.94%), NIP (32.99%), CNC (72.45%), and WL (55.44%) groups were similar (*χ^2^* = 737.823, df = 3, *p* < 0.001; Table 3). In the stratified analyses, all variables remained significant (all *p* < 0.001; Supplementary Table S3); these trends were consistent across female respondents, intellectual workers, individuals not involved in working in nutrition-related fields, disease-free individuals, individuals not on weight loss programs, and weekly snackers.

**Table 3:** Correct ranking rates for five food categories in different FOPL model groups (%).

Category	NC (*n* = 1,833)	NIP (*n* = 1,828)	CNC (*n* = 1,840)	WL (*n* = 1,802)	*p*
Total	69.94^a^	32.99^b^	72.45^a^	55.44^c^	<0.001
Beverages	71.63^a^	44.97^b^	76.25^c^	67.65^d^	<0.001
Potato chips	77.14^a^	55.20^b^	75.76^a^	75.69^a^	<0.001
Cookies	65.41^a^	13.13^b^	64.08^a^	36.96^c^	<0.001
Instant noodles	70.81^a^	42.40^b^	73.04^a^	45.89^c^	<0.001
Yoghurt	70.38^a^	37.42^b^	73.80^c^	31.30^d^	<0.001

^a, b, c, d^The same letter appearing for two groups denotes the absence of significant between-group differences, whereas different letters for different groups denote the presence of significant between-group differences (*p* < 0.05).

**Figure 3:**
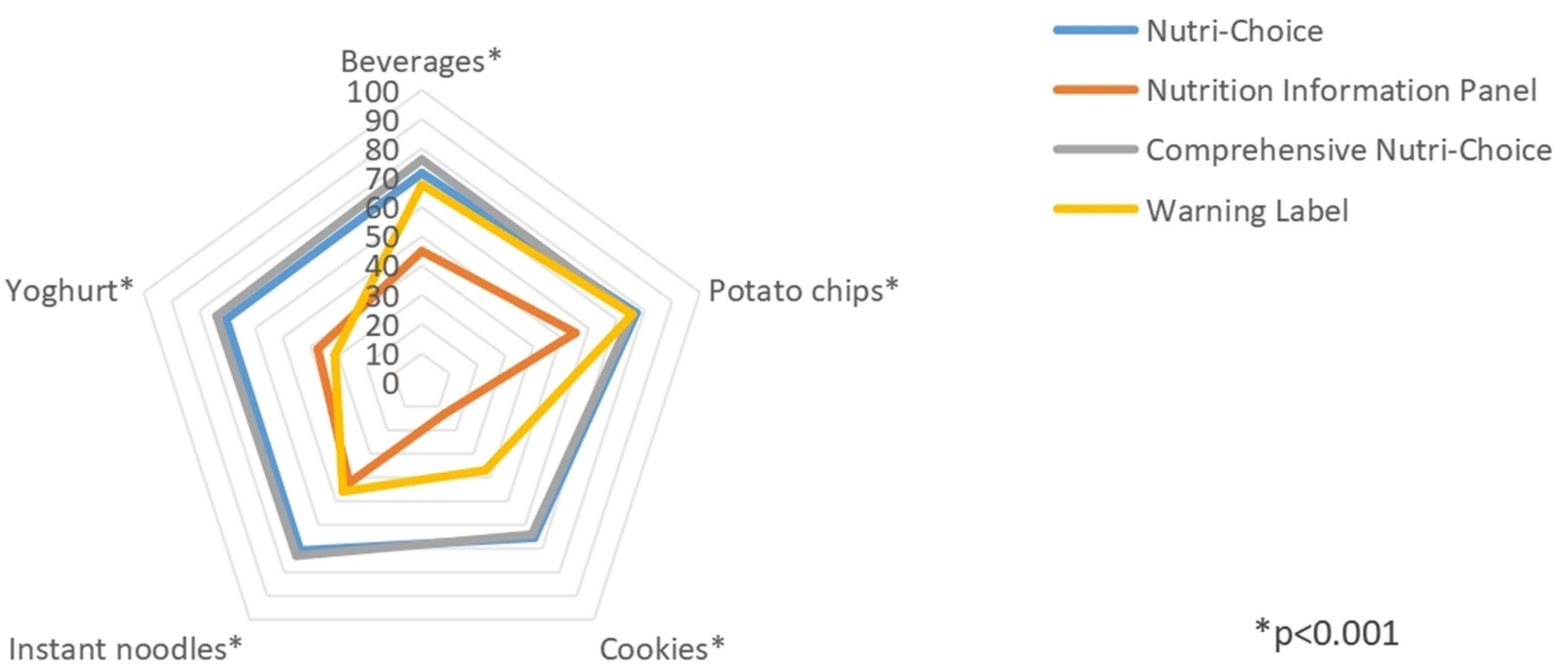
Correct ranking rates for five food categories in different FOPL model groups.

Figure 3 and Table 3 illustrate the percentages of respondents correctly ranking in the five food categories according to different FOPL models. In general, for all five food categories, the differences in correct ranking rates were significant among all four FOPL groups (all *p* < 0.001). The pairwise comparison analyses of beverage and yoghurt categories revealed that the correct ranking rate was significantly higher in the CNC group, compared to other FOPL groups (*p* < 0.05). NC group recorded the second highest correct ranking rates in these food categories, significantly different from WL and NIP groups. WL group performed better than NIP group in beverage category (67.65% vs. 44.97%, *p* < 0.05), but not in yoghurt category (31.30% vs. 37.42%, *p* < 0.05). For cookie and instant noodle categories, both NC and CNC groups had similar correct ranking rate (*p* > 0.05) and performed better than WL and NIP groups (*p* < 0.05). In addition, WL group recorded significantly higher correct ranking rate than NIP group (*p* < 0.05). For potato chip category, NIP group demonstrated a significantly lower correct ranking rate than other FOPL groups (*p* < 0.05). However, no significant difference observed among NC, CNC, and WL groups (*p* > 0.05).

### Effects of different FOPL models on the intention to purchase healthy foods

The analysis of the impact of different FOPL models on purchase intention indicated that very even distribution of total rates of intention to purchase healthy foods in the NC (80.09%), CNC (83.10%), NIP (75.11%), and WL (73.42%) groups (*χ^2^* = 63.360, df = 3, *p* < 0.001; Table 4). In the stratified analyses, all variables were significant (all *p* < 0.001; Table 4). Notably, among respondents aged 18–40 years, the intention rate was the highest in the WL group, the CNC group, the NC group, and finally, the NIP group. Moreover, among respondents with a monthly after-tax income of less than CN¥2,600, working an intellectual job, working as junior and senior employees, who were underweight and obese, with at least one serious disease, in the weight loss/shaping period, with a preference for snacking, and consuming snacks daily, the intention rates were higher in the NIP and CNC groups than in the WL and NC groups. The other variables differed similarly to the total accuracy rate (all *p* < 0.001; Supplementary Table S4).

**Table 4:** Rates of intention to make healthy purchases for different FOPL models in five food categories (%).

Category	NC (*n* = 1,833)	NIP (*n* = 1,828)	CNC (*n* = 1,840)	WL (*n* = 1,802)	*p*
Total	80.09^a^	75.11^b^	83.10^c^	73.42^b^	<0.001
Beverage	86.58^a^	82.99^b^	72.77^c^	86.74^a^	<0.001
Potato chips	88.38^a^	84.19^b^	89.13^a^	90.01^a^	<0.001
Cookies	68.69^a^	43.49^b^	69.84^a^	39.57^c^	<0.001
Instant noodles	73.65^a^	59.19^b^	78.26^c^	52.00^d^	<0.001
Yoghurt	75.01^a^	47.26^b^	78.37^c^	49.50^b^	<0.001

^a, b, c, d^The same letter appearing for two groups denotes the absence of significant between-group differences, whereas different letters for different groups signify the presence of significant between-group differences (*p* < 0.05).

**Figure 4:**
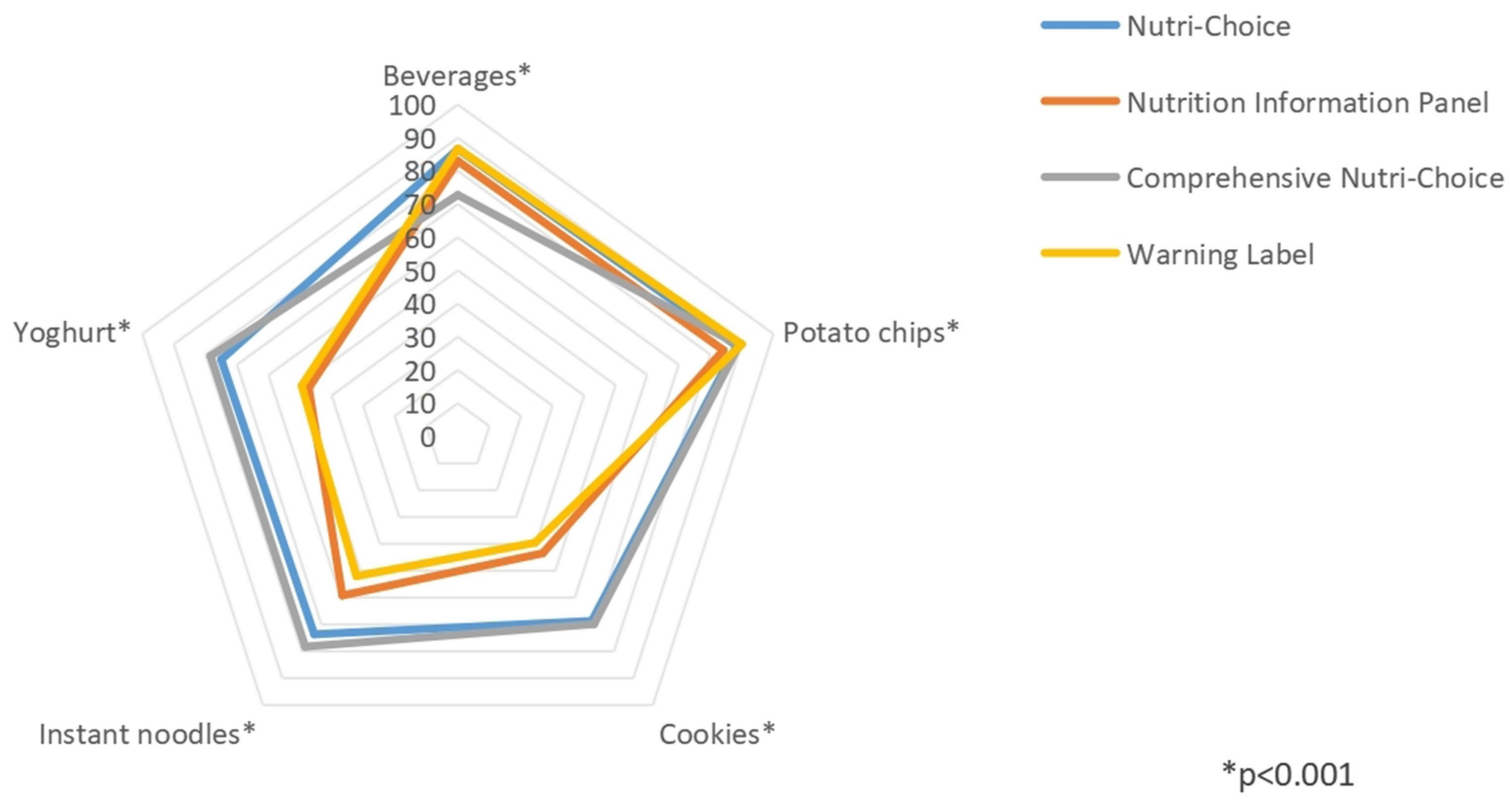
Rates of intention to make healthy purchases for different FOPL models in five food categories.

In general, the differences in the rates of intention to purchase healthy foods in all five categories of foods were significant across all four groups (all *p* < 0.001; Figure 4 and Table 4). In the pairwise comparison analysis, the rates for beverages were higher in the NC and WL groups than in the NIP group, and the rate was higher in the NIP group than in the CNC group (*p* < 0.05). For potato chips, the rates were higher in the NC, CNC, and WL groups than in the NIP group (*p* < 0.05). For cookies, the rates were higher in the NC and CNC groups than in the NIP group, and the rate was higher in the NIP group than in the WL group (*p* < 0.05). For instant noodles, the rate was the highest in the CNC group, followed by the NC group, the NIP group, and finally, the WL group (*p* < 0.05). Finally, for yoghurt, the rate was the highest in the CNC group, followed by the NC group, the WL group, and finally, the NIP group (*p* < 0.05).

## Discussion

China has a unique dietary culture, with habits strongly differing from those in other countries (22). Currently, although several teams in China have developed FOPL tailored specifically for the country’s market; the types of FOPLs they explored were limited and did not adequately investigate the FOPL types that are best suited to the national conditions of China (14, 17, 18). With an appropriate FOPL policy, the Chinese government may benefit the country’s population of 1.4 billion. Studies based on real-world or simulated shopping scenarios to evaluate the effectiveness of different FOPL models in the Chinese population have been scant. Our results provided a foundation for exploring FOPL’s applicability in China, as well as evidence to select an FOPL scheme best aligning with the country’s policy objectives and offering scientific evidence to countries experiencing a substantial China-like nutritional transition.

In our survey, a majority (88.03%) of respondents favored nutrition guidance labels. A study on FOPL preferences in 2024 conducted in Henan, China, reported that 81.51% of consumers favored FOPL implementation (23). Notably, in the current study, Shanghai residents demonstrated a slight increase in support for FOPL. This result is likely attributable to the elevated average income and education levels of Shanghai residents, who tend to prioritize dietary health (24), which may foster increased awareness and support for FOPL.

Our survey assessed respondents’ sensory perceptions of the four FOPL models: NC, NIP, CNC, and WL. Across all eight dimensions, NC, CNC, and WL scored higher than NIP. Notably, WL was particularly prominent in terms of impression and influence on purchasing. This may be because of the strong impact of WL deterring respondents from unhealthy foods high in sodium, sugar, and fat—a finding consistent with those reported previously (25–27). However, in Chinese culture, black is often associated with negativity, such as pain, misfortune, and bad luck, making it less appealing to Chinese consumers. Furthermore, positive messaging is generally more effective in communication than negative messaging (28).

Regarding nutrition ranking and intention to purchase healthier foods, NC and CNC outperformed WL and NIP. This result may be attributed to the simplicity, comprehensibility, and vibrant colors of NC and CNC. Color is pivotal in determining FOPL prominence to capture attention and improve perception regarding nutritional options (29, 30). Moreover, color combined with simple nutritional level descriptions of FOPL can enhance the understanding of the nutritional levels of foods (31). Thus, color-coded FOPL models such as NC and CNC, with their bold red and green contrasts, quickly draw consumers’ attention, encouraging them to consider food labels, in turn fostering healthier food selection.

In the present study, we employed NC and CNC with an ABCD system to indicate the nutritional levels explicitly, simplifying nutritional value assessment and enabling efficient choice of healthy options in contrast to NIP. Globally, FOPL models such as Nutri-score and the 5-Colour Nutrition Label, which also use eye-catching colors and hierarchical systems, have been effective (14, 32, 33). Notably, NS, recognized for its trustworthiness and user-friendliness, can effectively guide healthy food selection. In a comparative study across 12 countries (34), NS significantly increased correct choice rates for pizza, cake, and cereal by 47, 229, and 95%, respectively—surpassing the rates for other FOPL models. In a 1-year follow-up study, NS influenced the purchasing intentions of 42.9% of respondents, directing them toward healthier choices (35). These results align with our findings, confirming the validity of our research. Therefore, compared with the black and white WL, the visually pleasing, positive NC and CNC may be appealing and promotable among Chinese consumers.

Although both NC and CNC demonstrated comparable results in terms of consumer appeal and acceptance, our respondents completed NC more rapidly than CNC. This was likely due to the additional food-specific nutrient information included in CNC, beyond mere nutrient level grades, necessitating additional comprehension time. Furthermore, our analysis revealed that of the four FOPL models, NIP required the most time to comprehend. In other words, NIP was the most challenging to comprehend and accept—aligning with insights reported elsewhere (17, 36).

In this study, we used random questionnaire selection and RCT randomization to form groups. This approach effectively controlled for the potential confounding factors, further enhancing the comparability of data across different groups. Second, we included a large sample, encompassing a diverse range of age groups, economic statuses, and geographic regions through rigorous randomization, which guaranteed that our dataset was comprehensive and highly representative. Third, by examining four FOPL models on prepackaged foods, we provided a holistic assessment of public perception and preferences regarding FOPL. Our findings may guide the promotion of various healthy prepackaged foods in China.

This study, however, has some limitations. First, we only explored four FOPL models. Thus, additional studies exploring nutritional labels comprehensively are required. Second, all sorting activities related to shopping were performed online, while the simulated online shopping design was well executed, and we could not reflect the real-world shopping experience completely (e.g., actual buying decisions, physical product handling). Further research with experiments in real-world scenarios is warranted.

In summary, regardless of sex, age, educational and economic levels, and health conditions, our respondents favored accessing nutritional information via FOPL to guide their food choices. In particular, both NC and CNC, featuring colorful, simplified grading systems, effectively aided our respondents in more quickly comprehending food labeling, make healthier food choices, and gain a deeper sensory impression. In terms of information-processing time, NC was superior to CNC. Therefore, we recommend implementing NC-positive labels on prepackaged foods and actively promoting them in China.

## Data Availability

The raw data supporting the conclusions of this article will be made available by the authors, without undue reservation.
