# Supplementary material for: Impact of different front-of-package labeling models on food choices among residents in Shanghai, China: a randomized controlled trial
Source: Front Nutr. 2025 Jul 4;12:1594807. doi: 10.3389/fnut.2025.1594807 (PMC12272224; doi:10.3389/fnut.2025.1594807)
Supplement: Supplementary file 1 [file Table_1.docx]

**Table S1** Factors influencing attitudes towards FOPL (%)

| Characteristic | Use FOPL to distinguish whether food is nutritious (N = 7303) | | | *p* (chi-square test) |
| --- | --- | --- | --- | --- |
|  | Agree (n = 6429) | Neither agree nor disagree (n = 806) | Disagree (n = 68) |  |
| **Total** | 88.03 | 11.04 | 0.93 |  |
| **Sex** |  |  |  | 0.025 |
| Male | 87.78 | 10.97 | 1.17 |  |
| Female | 88.27 | 11.10 | 0.33 |  |
| **Age (years)** |  |  |  | 0.120 |
| [6,18) | 87.61 | 11.75 | 0.64 |  |
| [18, 40) | 88.91 | 10.10 | 0.99 |  |
| [40,60) | 88.88 | 10.02 | 1.10 |  |
| ≥60 | 86.79 | 11.97 | 1.24 |  |
| **Education** |  |  |  | 0.007 |
| Junior high school or lower | 87.36 | 11.76 | 0.87 |  |
| High school (general/vocational/secondary/technical school) or junior college | 87.33 | 11.90 | 0.78 |  |
| Undergraduate and above | 89.76 | 9.04 | 1.20 |  |
| **Average monthly earnings after taxes* (CN¥)** |  |  |  | 0.000 |
| Less than 2600 | 86.70 | 12.20 | 1.11 |  |
| 2600-6000 | 87.16 | 12.04 | 0.80 |  |
| 6,000 and above | 89.65 | 9.03 | 1.32 |  |
| **Occupation** |  |  |  | 0.162 |
| Intellectual | 89.07 | 9.77 | 1.16 |  |
| Manual labour | 88.02 | 10.93 | 1.05 |  |
| Student | 87.56 | 11.75 | 0.68 |  |
| **Job title** |  |  |  | 0.041 |
| None | 87.77 | 11.31 | 0.92 |  |
| Junior | 90.16 | 8.59 | 1.25 |  |
| Intermediate | 89.10 | 9.24 | 1.66 |  |
| Senior | 87.62 | 11.43 | 0.95 |  |
| **Engaged in nutrition-, food-, or medicine-related industries** |  |  |  |  |
| Yes | 87.94 | 11.19 | 0.87 | 0.109 |
| No | 88.84 | 9.64 | 1.52 |  |
| **Body mass index** |  |  |  | 0.834 |
| Thin | 88.68 | 9.75 | 1.57 |  |
| Normal | 88.08 | 11.07 | 0.85 |  |
| Overweight | 88.03 | 11.00 | 0.97 |  |
| Obesity | 87.03 | 11.79 | 1.18 |  |
| **At least one serious disease (e.g. diabetes, anaemia, thyroid disorder, and heart disease)** |  |  |  | 0.210 |
| Yes | 89.44 | 8.91 | 1.65 |  |
| No | 87.97 | 11.13 | 0.90 |  |
| **Weight loss/shaping** |  |  |  | 0.259 |
| Yes | 89.81 | 9.25 | 0.94 |  |
| No | 87.83 | 11.24 | 0.93 |  |
| **Snacking preference** |  |  |  |  |
| Like | 87.97 | 11.26 | 0.77 |  |
| Neither like nor dislike | 87.77 | 11.26 | 0.97 |  |
| Dislike | 88.70 | 10.17 | 1.14 |  |
| **Snacking frequency** |  |  |  | 0.007 |
| Daily | 84.74 | 14.36 | 0.90 |  |
| 4–6 times/week | 89.62 | 9.27 | 1.11 |  |
| 1–3 times/week | 87.61 | 11.59 | 0.80 |  |
| Less than 1 time/week | 88.77 | 10.20 | 1.03 |  |
| **Living with any children aged <15 years** |  |  |  | 0.012 |
| Yes | 89.83 | 9.39 | 0.77 |  |
| No | 87.32 | 11.68 | 0.99 |  |

* This part of the data does not include students, the remaining total number is 4521

**Table S2** Respondent FOPL concurrence rates in terms of sensory perceptions in all and different FOPL model groups (%)

| Characteristic | Total (N = 7303) | Label 1: NC (n = 1833) | Label 2: NIP (n = 1828) | Label 3: CNC (n = 1840) | Label 4: WL (n = 1802) | *p* (Kruskal–Wallis test) |
| --- | --- | --- | --- | --- | --- | --- |
| **Total** | 54.03 | 57.01^a^ | 43.33^b^ | 58.04^a^ | 57.77^a^ | <0.001 |
| **Sex** |  |  |  |  |  |  |
| Male | 54.20 | 57.90^a^ | 42.00^b^ | 57.60^a^ | 59.30^a^ | <0.001 |
| Female | 53.90 | 56.20^a^ | 44.60^b^ | 58.40^a^ | 56.20^a^ | <0.001 |
| **Age (years)** |  |  |  |  |  |  |
| [6,18) | 54.60 | 58.30^a^ | 44.00^b^ | 59.70^a^ | 56.60^a^ | <0.001 |
| [18, 40) | 55.10 | 56.60^a^ | 41.40^b^ | 61.60^a^ | 60.60^a^ | <0.001 |
| [40,60) | 51.60 | 51.40^a,b^ | 44.40^b^ | 52.80^a^ | 58.00^a^ | 0.003 |
| ≥60 | 54.20 | 61.60^a^ | 43.20^b^ | 56.60^a^ | 56.30^a^ | <0.001 |
| **Education** |  |  |  |  |  |  |
| Junior high school or lower | 56.10 | 62.00^a^ | 45.30^b^ | 59.10^a^ | 58.10^a^ | <0.001 |
| High school (general/vocational/secondary/technical school) or junior college | 52.90 | 55.50^a^ | 43.90^b^ | 56.20^a^ | 56.40^a^ | <0.001 |
| Undergraduate and above | 53.00 | 53.50^a^ | 40.20^b^ | 59.10^a^ | 59.10^a^ | <0.001 |
| **Average monthly earnings after taxes* (CN¥)** |  |  |  |  |  |  |
| Less than 2600 | 51.70 | 60.60^a^ | 39.60^b^ | 54.30^a^ | 51.90^a,b^ | 0.017 |
| 2600-6000 | 53.00 | 57.10^a^ | 42.30^b^ | 53.30^a^ | 59.50^a^ | <0.001 |
| 6,000 and above | 54.10 | 53.50^a^ | 43.40^b^ | 60.90^a^ | 59.00^a,b^ | <0.001 |
| **Occupation** |  |  |  |  |  |  |
| Intellectual | 52.70 | 52.10^a^ | 40.80^b^ | 60.60^b^ | 57.60^a,b^ | <0.001 |
| Manual labour | 53.60 | 57.00^a^ | 43.30^b^ | 55.50^a^ | 58.90^a^ | <0.001 |
| Student | 55.10 | 59.10^a^ | 44.60^b^ | 59.90^a^ | 56.60^a^ | <0.001 |
| **Job title** |  |  |  |  |  |  |
| None | 53.20 | 56.90^a^ | 42.80^b^ | 56.00^a^ | 57.10^a^ | <0.001 |
| Junior | 54.40 | 51.00^a,b^ | 46.00^b^ | 60.90^a^ | 58.90^a^ | 0.023 |
| Intermediate | 53.70 | 55.90^a^ | 39.90^b^ | 57.40^a^ | 63.20^a^ | <0.001 |
| Senior | 49.50 | 39.10^a,b^ | 33.30^b^ | 56.00^a,b^ | 63.60^a^ | 0.087 |
| **Engaged in nutrition-, food-, or medicine-related industries** |  |  |  |  |  |  |
| Yes | 52.30 | 48.60^a,b^ | 43.20^b^ | 60.30^b^ | 57.60^a,b^ | 0.003 |
| No | 54.20 | 57.90^a^ | 43.30^b^ | 57.80^a^ | 57.80^a^ | <0.001 |
| **Body mass index** |  |  |  |  |  |  |
| Thin | 55.70 | 61.60^a^ | 49.40^a^ | 54.90^a^ | 56.20^a^ | 0.473 |
| Normal | 54.50 | 57.60^a^ | 43.30^b^ | 59.50^a^ | 57.10^a^ | <0.001 |
| Overweight | 54.30 | 57.00^a^ | 45.00^b^ | 55.10^a^ | 61.40^a^ | 0.001 |
| Obesity | 46.90 | 45.50^a,b^ | 32.40^b^ | 56.90^a^ | 53.30^a^ | <0.001 |
| **At least one serious disease (e.g. diabetes, anaemia, thyroid disorder, and heart disease)** |  |  |  |  |  |  |
| Yes | 50.50 | 52.10^a^ | 45.90^a^ | 50.60^a^ | 53.40^a^ | 0.816 |
| No | 54.20 | 57.20^a^ | 43.20^b^ | 58.40^a^ | 58.00^a^ | <0.001 |
| **Weight loss/shaping** |  |  |  |  |  |  |
| Yes | 52.40 | 53.30^a^ | 37.60^b^ | 59.30^a^ | 59.60^a^ | <0.001 |
| No | 54.20 | 57.40^a^ | 44.00^b^ | 57.90^a^ | 57.60^a^ | <0.001 |
| **Snacking preference** |  |  |  |  |  |  |
| Like | 54.70 | 58.80^a^ | 43.40^b^ | 59.20^a^ | 57.70^a^ | <0.001 |
| Neither like nor dislike | 53.80 | 56.00^a^ | 43.70^b^ | 56.60^a^ | 58.40^a^ | <0.001 |
| Dislike | 53.40 | 55.90^a^ | 42.30^b^ | 59.10^a^ | 56.70^a^ | <0.001 |
| **Snacking frequency** |  |  |  |  |  |  |
| Daily | 48.60 | 48.90^b^ | 40.20^b^ | 52.40^a^ | 53.30^a^ | 0.035 |
| 4–6 times/week | 57.10 | 61.40^a^ | 44.50^b^ | 61.90^a^ | 60.40^a^ | <0.001 |
| 1–3 times/week | 54.40 | 57.60^a^ | 44.40^b^ | 57.00^a^ | 58.40^a^ | <0.001 |
| Less than 1 time/week | 52.40 | 53.60^a^ | 41.30^b^ | 59.30^a^ | 55.80^a^ | <0.001 |
| **Living with any children aged <15 years** |  |  |  |  |  |  |
| Yes | 55.10 | 59.90^a^ | 41.60^b^ | 60.80^a^ | 58.00^a^ | <0.001 |
| No | 53.60 | 55.90^a^ | 44.00^b^ | 56.90^a^ | 57.70^a^ | <0.001 |

*These data do not include students; the remaining total number is 4521.

a, b, c, d: The same letter appearing for two groups denotes the absence of significant between-group differences, whereas different letters for different groups denote the presence of significant between-group differences (*p* < 0.05).

**Table S3**  Correct ranking rates in all and different FOPL model groups (%)

| Characteristic | Total (N = 7303) | Label 1: NC (n = 1833) | Label 2: NIP (n = 1828) | Label 3: CNC (n = 1840) | Label 4: WL (n = 1802) | *p* (chi-square test) |
| --- | --- | --- | --- | --- | --- | --- |
| **Total** | 57.74 | 69.94^a^ | 32.99^b^ | 72.45^a^ | 55.44^c^ | <0.001 |
| **Sex** |  |  |  |  |  |  |
| Male | 57.36 | 69.65^a^ | 32.80^b^ | 73.60^a^ | 54.00^b^ | <0.001 |
| Female | 58.10 | 70.20^a^ | 33.20^b^ | 71.40^a^ | 51.90^c^ | <0.001 |
| **Age (years)** |  |  |  |  |  |  |
| [6,18) | 56.90 | 70.00^a^ | 31.80^b^ | 73.50^a^ | 52.10^b^ | <0.001 |
| [18, 40) | 61.50 | 76.50^a^ | 33.60^b^ | 76.70^a^ | 58.80^b^ | <0.001 |
| [40,60) | 56.20 | 67.50^a^ | 31.20^b^ | 68.60^a^ | 56.90^b^ | <0.001 |
| ≥60 | 56.40 | 64.10^a^ | 36.40^b^ | 69.70^a^ | 56.00^b^ | <0.001 |
| **Education** |  |  |  |  |  |  |
| Junior high school or lower | 55.40 | 65.60^a^ | 34.80^b^ | 69.30^a^ | 51.70^b^ | <0.001 |
| High school (general/vocational/secondary/technical school) or junior college | 58.10 | 69.60^a^ | 31.20^b^ | 74.40^a^ | 57.50^b^ | <0.001 |
| Undergraduate and above | 60.10 | 75.30^a^ | 33.20^b^ | 73.90^a^ | 57.60^b^ | <0.001 |
| **Average monthly earnings after taxes* (CN¥)** |  |  |  |  |  |  |
| Less than 2600 | 49.40 | 59.60^a^ | 28.80^b^ | 55.90^a^ | 52.90^b^ | <0.001 |
| 2600-6000 | 56.20 | 64.70^a^ | 34.50^b^ | 68.20^a^ | 57.40^b^ | <0.001 |
| 6,000 and above | 60.60 | 74.10^a^ | 33.70^b^ | 76.80^a^ | 58.40^b^ | <0.001 |
| **Occupation** |  |  |  |  |  |  |
| Intellectual | 58.00 | 73.00^a^ | 31.10^b^ | 74.80^a^ | 54.20^c^ | <0.001 |
| Manual labour | 57.50 | 67.40^a^ | 34.60^b^ | 69.40^a^ | 58.80^b^ | <0.001 |
| Student | 57.90 | 71.50^a^ | 21.00^b^ | 75.10^a^ | 52.20^b^ | <0.001 |
| **Job title** |  |  |  |  |  |  |
| None | 57.20 | 67.80^a^ | 34.50^b^ | 69.10^a^ | 56.70^b^ | <0.001 |
| Junior | 59.50 | 71.00^a^ | 27.60^b^ | 75.70^a^ | 64.40^b^ | <0.001 |
| Intermediate | 58.50 | 72.30^a^ | 34.30^b^ | 72.20^a^ | 58.00^b^ | <0.001 |
| Senior | 55.20 | 69.60^a^ | 37.50^b^ | 84.00^a^ | 36.40^b^ | <0.001 |
| **Engaged in nutrition-, food-, or medicine-related industries** |  |  |  |  |  |  |
| Yes | 57.40 | 70.90^a^ | 31.60^b^ | 73.50^a^ | 54.70^b^ | <0.001 |
| No | 57.80 | 69.80^a^ | 33.20^b^ | 72.30^a^ | 55.50^c^ | <0.001 |
| **Body mass index** |  |  |  |  |  |  |
| Thin | 56.90 | 68.60^a^ | 31.20^b^ | 68.30^a^ | 57.50^b^ | <0.001 |
| Normal | 58.10 | 70.10^a^ | 32.80^b^ | 74.00^a^ | 55.10^b^ | <0.001 |
| Overweight | 56.80 | 69.00^a^ | 33.50^b^ | 68.30^a^ | 57.80^b^ | <0.001 |
| Obesity | 58.00 | 73.70^a^ | 34.20^b^ | 76.10^a^ | 49.50^b^ | <0.001 |
| **At least one serious disease (e.g. diabetes, anaemia, thyroid disorder, and heart disease)** |  |  |  |  |  |  |
| Yes | 55.10 | 67.60^a^ | 28.40^b^ | 68.20^a^ | 54.80^b^ | <0.001 |
| No | 57.90 | 70.00^a^ | 33.20^b^ | 72.60^a^ | 55.50^c^ | <0.001 |
| **Weight loss/shaping** |  |  |  |  |  |  |
| Yes | 58.60 | 73.30^a^ | 31.20^b^ | 75.10^a^ | 54.80^b^ | <0.001 |
| No | 57.60 | 69.50^a^ | 33.20^b^ | 72.20^a^ | 55.50^c^ | <0.001 |
| **Snacking preference** |  |  |  |  |  |  |
| Like | 57.50 | 69.00^a^ | 34.60^b^ | 73.40^a^ | 52.60^b^ | <0.001 |
| Neither like nor dislike | 57.90 | 70.40^a^ | 31.70^b^ | 72.10^a^ | 55.60^b^ | <0.001 |
| Dislike | 57.90 | 70.70^a^ | 32.50^b^ | 71.30^a^ | 59.60^b^ | <0.001 |
| **Snacking frequency** |  |  |  |  |  |  |
| Daily | 48.80 | 54.90^a^ | 28.90^b^ | 66.30^c^ | 45.10^b^ | <0.001 |
| 4–6 times/week | 59.60 | 71.30^a^ | 36.20^b^ | 75.30^a^ | 54.90^b^ | <0.001 |
| 1–3 times/week | 58.40 | 70.70^a^ | 32.50^b^ | 71.70^a^ | 57.20^c^ | <0.001 |
| Less than 1 time/week | 58.90 | 74.60^a^ | 32.40^b^ | 74.40^a^ | 57.30^b^ | <0.001 |
| **Living with any children aged <15 years** |  |  |  |  |  |  |
| Yes | 59.50 | 70.20^a^ | 34.00^b^ | 76.50^a^ | 56.20^b^ | <0.001 |
| No | 57.00 | 69.90^a^ | 32.60^b^ | 70.70^a^ | 55.20^b^ | <0.001 |

*These data do not include students; the remaining total number is 4521.

a, b, c, d: The same letter appearing for two groups denotes the absence of significant between-group differences, whereas different letters for different groups denote the presence of significant between-group differences (*p* < 0.05).

**Table S4** Rates of intention to make healthy purchases in all and different FOPL model groups (%)

| Characteristic | Total (N = 7303) | Label 1: NC (n = 1833) | Label 2 NIP (n = 1828) | Label 3: CNC (n = 1840) | Label 4: WL (n = 1802) | *p* (chi-square test) |
| --- | --- | --- | --- | --- | --- | --- |
| **Total** | 78.00 | 80.09^a^ | 75.11^b^ | 83.10^c^ | 73.42^b^ | <0.001 |
| **Sex** |  |  |  |  |  |  |
| Male | 77.30 | 79.20^a^ | 74.40^b^ | 83.60^c^ | 72.00^b^ | <0.001 |
| Female | 78.60 | 80.90^a^ | 75.80^b^ | 82.60^c^ | 74.80^b^ | <0.001 |
| **Age (years)** |  |  |  |  |  |  |
| [6,18) | 76.30 | 77.40^a^ | 73.20^b^ | 83.90^c^ | 70.70^b^ | <0.001 |
| [18, 40) | 80.40 | 84.40^a^ | 76.00^b^ | 85.30^c^ | 76.10^d^ | <0.001 |
| [40,60) | 77.70 | 80.30^a^ | 76.10^b^ | 82.10^c^ | 71.70^b^ | <0.001 |
| ≥60 | 78.30 | 79.90^a^ | 76.60^b^ | 80.00^c^ | 77.00^b^ | <0.001 |
| **Education** |  |  |  |  |  |  |
| Junior high school or lower | 76.40 | 76.50^a^ | 75.00^b^ | 82.20^c^ | 71.70^b^ | <0.001 |
| High school (general/vocational/secondary/technical school) or junior college | 77.90 | 80.90^a^ | 73.80^b^ | 83.40^c^ | 73.20^b^ | <0.001 |
| Undergraduate and above | 80.00 | 83.00^a^ | 77.10^b^ | 83.90^c^ | 75.80^b^ | <0.001 |
| **Average monthly earnings after taxes* (CN¥)** |  |  |  |  |  |  |
| Less than 2600 | 71.80 | 66.10^a^ | 73.00^b^ | 74.00^a^ | 74.00^b^ | <0.001 |
| 2600-6000 | 78.80 | 82.50^a^ | 76.60^b^ | 81.80^c^ | 74.40^b^ | <0.001 |
| 6,000 and above | 81.00 | 82.40^a^ | 77.10^b^ | 84.70^c^ | 76.30^b^ | <0.001 |
| **Occupation** |  |  |  |  |  |  |
| Intellectual | 80.90 | 83.60^a^ | 76.10^b^ | 85.80^a^ | 78.30^b^ | <0.001 |
| Manual labour | 77.90 | 79.80^a^ | 76.60^b^ | 80.90^c^ | 74.00^b^ | <0.001 |
| Student | 76.70 | 78.90^a^ | 72.80^b^ | 84.50^c^ | 70.40^b^ | <0.001 |
| **Job title** |  |  |  |  |  |  |
| None | 78.30 | 79.90^a^ | 76.30^b^ | 81.80^c^ | 74.80^b^ | <0.001 |
| Junior | 79.10 | 80.70^a^ | 77.30^b^ | 81.10^a^ | 77.30^b^ | <0.001 |
| Intermediate | 80.10 | 84.70^a^ | 77.80^b^ | 83.00^c^ | 75.30^b^ | <0.001 |
| Senior | 81.00 | 82.60^a^ | 66.70^b^ | 100.00^a^ | 75.80^b^ | <0.001 |
| **Engaged in nutrition-, food-, or medicine-related industries** |  |  |  |  |  |  |
| Yes | 76.60 | 77.70^a^ | 76.30^b^ | 81.50^c^ | 70.30^b^ | <0.001 |
| No | 78.10 | 80.30^a^ | 75.00^b^ | 83.30^c^ | 73.70^b^ | <0.001 |
| **Body mass index** |  |  |  |  |  |  |
| Thin | 76.40 | 74.40^a^ | 66.20^b^ | 90.20^a^ | 74.00^b^ | <0.001 |
| Normal | 78.50 | 80.50^a^ | 75.10^b^ | 84.40^c^ | 74.20^b^ | <0.001 |
| Overweight | 77.30 | 79.80^a^ | 77.40^b^ | 78.70^c^ | 72.80^b^ | <0.001 |
| Obesity | 75.20 | 81.80^a^ | 71.20^b^ | 81.70^a^ | 66.70^b^ | <0.001 |
| **At least one serious disease (e.g. diabetes, anaemia, thyroid disorder, and heart disease)** |  |  |  |  |  |  |
| Yes | 78.20 | 76.10^a^ | 77.00^b^ | 81.20^a^ | 78.10^b^ | <0.001 |
| No | 77.90 | 80.20^a^ | 75.00^b^ | 83.20^c^ | 73.20^b^ | <0.001 |
| **Weight loss/shaping** |  |  |  |  |  |  |
| Yes | 76.70 | 77.90^a^ | 73.10^b^ | 84.20^a^ | 71.80^b^ | <0.001 |
| No | 78.10 | 80.30^a^ | 75.30^b^ | 83.00^c^ | 73.60^b^ | <0.001 |
| **Snacking preference** |  |  |  |  |  |  |
| Like | 76.60 | 77.30^a^ | 74.50^b^ | 83.60^a^ | 70.60^b^ | <0.001 |
| Neither like nor dislike | 78.10 | 81.60^a^ | 74.30^b^ | 82.30^c^ | 73.90^b^ | <0.001 |
| Dislike | 80.10 | 82.30^a^ | 77.90^b^ | 84.10^c^ | 77.00^b^ | <0.001 |
| **Snacking frequency** |  |  |  |  |  |  |
| Daily | 71.50 | 66.80^a^ | 73.00^b^ | 78.40^a^ | 66.80^b^ | <0.001 |
| 4–6 times/week | 77.90 | 79.70^a^ | 77.10^b^ | 84.00^c^ | 70.80^b^ | <0.001 |
| 1–3 times/week | 74.20 | 81.50^a^ | 72.70^b^ | 83.20^c^ | 74.20^b^ | <0.001 |
| Less than 1 time/week | 81.20 | 84.40^a^ | 78.90^b^ | 84.60^c^ | 77.80^b^ | <0.001 |
| **Living with any children aged <15 years** |  |  |  |  |  |  |
| Yes | 79.00 | 80.40^a^ | 76.50^b^ | 85.60^c^ | 72.60^b^ | <0.001 |
| No | 77.50 | 80.00^a^ | 74.60^b^ | 82.00^c^ | 73.70^b^ | <0.001 |

*These data do not include students; the remaining total number is 4521.

a, b, c, d: The same letter appearing for two groups denotes the absence of significant between-group differences, whereas different letters for different groups denote the presence of significant between-group differences (*p* < 0.05).
